# Antioxidant, Total Phenolic Content and Cytotoxicity Evaluation of Selected Malaysian Plants

**DOI:** 10.3390/molecules16043433

**Published:** 2011-04-21

**Authors:** Suhailah Wasman Qader, Mahmood Ameen Abdulla, Lee Suan Chua, Nigar Najim, Mazatulikhma Mat Zain, Salehhuddin Hamdan

**Affiliations:** 1 Department of Biological Science, Faculty of Biosciences and Bioengineering, University of Technology Malaysia, 81310, UTM Skudai, Johor, Malaysia; E-Mail: bmbsha@yahoo.com (S. H.); 2 Department of Molecular Medicine, Faculty of Medicine, University of Malaya, 50603, Kuala Lumpur, Malaysia; E-Mail: mahmood955@yahoo.com (M.A.A.); 3 Metabolites Profiling Laboratory, Chemical Engineering Pilot Plant, University Technology Malaysia, 81310, UTM Skudai, Johor, Malaysia; E-Mail: chualeesuan@yahoo.com (L.S.C.); 4 Tissue Culture Research Laboratory, Centre of Synthesis and Chemical Biology, Institute of Science, University Technology MARA, 40450, Shah Alam, Malaysia; E-Mails: nigar@salam.uitm.edu.my (N.N.); maizatul@salam.uitm.edu.my (M.M.Z.)

**Keywords:** antioxidant activity, total phenolic content, cytotoxicity, Malaysian plants

## Abstract

Aqueous and ethanol extracts of different traditional Malaysian plants (*Polygonum minus*, *Andrographis paniculata, Curcuma xanthorrhiza*, *Momordica charantia* and *Strobilanthes crispus)* were evaluated for their antioxidant properties, total phenolic content and cytotoxic activity. Antioxidant activity was evaluated by using 1,1-diphenyl-1-picrylhydrazyl (DPPH) and ferric reducing antioxidant power (FRAP) assays. The results showed that ethanol extracts contain high antioxidant activities compared to aqueous extracts. The findings exhibited a strong correlation between antioxidant activity and the total phenol contents. In addition, all the plant extracts showed non-toxic effects against a normal human lung fibroblast cell line (Hs888Lu). Although traditionally aqueous extracts are used, we determined that ethanol extracts usually achieved better activity in the assays.

## 1. Introduction

There are various studies emphasizing that free radicals contribute to the development of many diseases, including hemorrhagic shock, arthritis, ageing, atherosclerosis, ischemia, Alzheimer and Parkinson’s disease, gastrointestinal disorders, tumor promotion and carcinogenesis [1]. Antioxidants are substances that play an important role in delaying or preventing degenerative diseases caused by oxidative damage of living cell components caused by free radicals [2]. Synthetic antioxidants such as butylated hydroxytoluene (BHT), butylated hydroxyanisole (BHA), *tert*-butylhydroquinone (TBHQ) and propyl gallate (PG) have been used over the years, however they are being examined for possible toxicity. Hence, there are intensive studies on natural polyphenolic antioxidants derived from plants to replace the synthetic antioxidants.

Malaysian plants have been widely used because of their prized aromas and tastes, which add variety and flavor to foodstuffs. Traditionally, many of these plants are also used to treat different human ailments. On the other hand, research has proven that some of the plants are toxic to humans and animals due to the presence of certain compounds [3]. Examination of plant extracts for potential toxicity is considered an important step in evaluating their suitability for commercial application. Thus, five Malaysian plants were selected to evaluate their bio-activities in different assays: *Polygonum minus*, locally named ‘kesum’ in Malay, is a plant having a sweet and pleasant aroma. It comes from the family Polygonaceae [4]. Traditionally, *P. minus* is used to treat stomach problems. Relatively few pharmacological studies have been conducted on *P. minus*, which have indicated antimicrobial and cytotoxic activity against HeLa (human cervical carcinoma) [5]. *Andrographis paniculata* is a medicinal plant, commonly known as king of bitters and named locally ‘hempedu bumi’ in Malaysia. It belongs to the family Acanthaceae. Traditionally, its leaves and roots have been used for different medicinal purposes in Asia and Europe [6]. Pharmacological research has demonstrated that *A. paniculata* possesses antimicrobial activity [7], antiviral properties [8], hepatoprotective and antioxidant [9], anti-diabetic [10], antihyperglycaemic [11] and antiangiogenic activity [12], in addition to anti inflammatory properties [13], and it is used in the treatment of upper respiratory tract infections [14]. *Curcuma xanthorrhiza* (temu lawak) from the family Zingeberaceae has been traditionally used to treat stomach diseases, liver disorders, constipation, bloody diarrhea, dysentery, children’s fevers, hemorrhoids, and skin eruptions [15]. Pharmacologically is has been reported that *Curcuma* has antimicrobial [15], anti-metastatic [16] and anti-cancer activities [17]. *Strobilanthes crispus* from the Acanthaceae family is known as ‘pecah kaca’ or ‘jin batu’ in Malaysia as well as ‘daun peach beling’ in Jakarta. It is widely used in traditional medicine as an anti-diabetic, diuretic, antilytic and laxative [18]. *S. crispus* has been proved scientifically to have antihyperglysemic, hypolipidemic and antioxidant enzyme effects [19], anti-AIDS and anticancer activity [20], as well as to provide possible protection against lipid peroxidation and DNA damage induced by iron nitrilotriacetate [21]. *Momordica charantia*, known as bitter melon or bitter gourd (family: Cucurbitaceae), is an herb widely grown in tropical countries like Malaysia and India. It is commonly eaten as a vegetable and has been proven to have antibacterial [22], antmalarial [23], anti-ulcer [24], antidiabetic [25], and potential hypocholesterolemic and anti-oxidant activities [26]. This study was undertaken to assess different Malaysian plants for their antioxidant activity and total phenolic content, as well as their potential *in vitro* toxicity.

## 2. Results and Discussions

### 2.1. Evaluation of DPPH Scavenging Activities

DPPH free radical scavenging activity of aqueous and ethanol extracts were evaluated to determine their antioxidant properties. The results are presented in Table 1.

**Figure 1:**
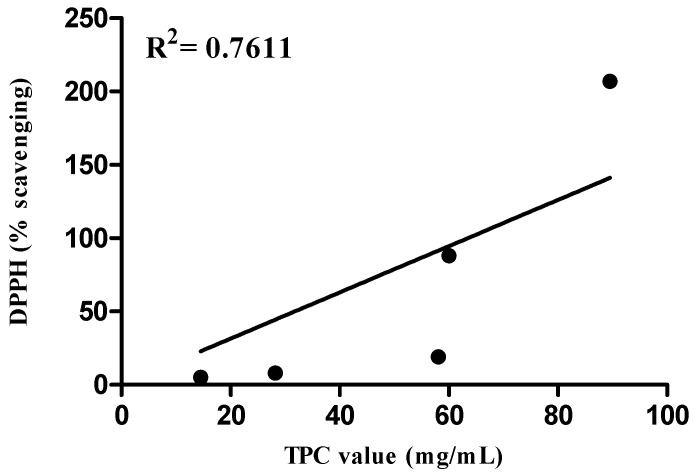
Correlation between TPC and DPPH scavenging percentage.

The DPPH free radical scavenging results of the positive control and plant extracts are expressed as a percentage of inhibition. Based on the values calculated from the linearity curves, the ethanol extracts showed a higher scavenging percentage than the aqueous extracts. As previously shown by Othman and co-workers [27], the solvent significantly influences the measurement of antioxidant properties of an extract. Furthermore, it has been shown by Scalzo and coworkers [28] and Giorgi and coworkers [29] that there is a different correlation between antioxidant activity and TPC. Our findings showed a positive correlation between TPC and DPPH for ethanolic extract, R2 = 0.7611 (Figure 1). Just as previously reported by Gorinstein and coworkers [30], a positive correlation between DPPH and TPC was shown. Since *P*. *minus* represented to be the best traditional plant for its phenolic content, so it is postulated that it woud represent the major source of antioxidant capacity. The results also indicated that there is no significant difference between the DPPH free radical scavenging capacity of *Polygonum minus* and the positive control (gallic acid). This is supported by Faujan [31], who demonstrated that amongst five water aromatic herb extracts *P*. *minus* showed the best antioxidant activities. Hence, we can conclude that *P. minus* may act as a potential source of antioxidants.

### 2.2. Ferric Reducing Antioxidant Power Assay (FRAP)

The reduction capacity of the tested Malaysian plants is also indicated in Table 1. It is clear that were significantly differences between the plants’ reducing powers. 

**Figure 2:**
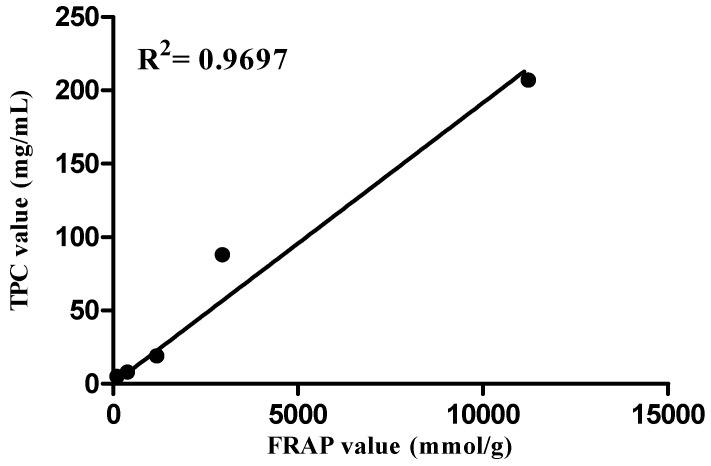
Correlation between total phenolic content and FRAP reducing power.

The FRAP values 11220 (± 0.1) and 849.33 (± 0.32) mmol/g of *Polygonum minus* showed the highest reducing power for both ethanol and aqueous extract, respectively. The results obtained by Faujan [31] showed that water extract of *P*. *minus* has the highest ability to reduce Fe(III) and was not significantly different with BHA. It is also found that there is a high correlation between TPC and FRAP, R^2^ = 0.9697 (Figure 2). Similarly, it has been proven that there is positive relation with TPC [32] and DPPH [33].

### 2.3. Total Phenolic Content (TPC)

The total phenolic content of each plant was shown in Table 1. The content of phenolics was significantly different between the plants; it ranges from 5 (± 0.002) to 207 (± 0.011) mg/g. *Polygonum minus* was found to have the highest TPC values 55.5 (± 0.002) mg/g and 207 (± 0.011) mg/g in both aqueous and ethanol extract, respectively. This value represents that 1 mg of plant extract contains amount of phenol equivalent to about 55.5 and 207 mg of pure gallic acid [34]. It is seen from Table 1 that the TPC for most of the ethanolic extracts were higher than those of the corresponding aqueous extracts. This is in agreement with the findings of Ling and co-workers [35] that predicted higher TPCs in ethanol extracts compare to aqueous extracts. In addition, Moure [36] demonstrated that high polarity of solvent yields high amounts of polyphenolics. Therefore, the significant TPC differences of this study may be attributable to the solvents of the extracts.

### 2.4. Cytotoxicity

**Figure 3:**
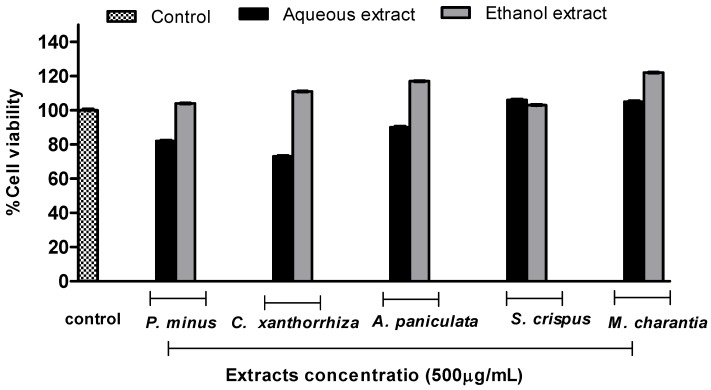
Cytotoxic activity of five Malaysian plants in normal lung (Hs888Lu) cell line at concentration of 500 µg/mL and 24 hr exposure time.

The results of cytotoxic activity of selected Malaysian plants were summarized in Figure 3, and were expressed versus percentage of the value observed with no plant treatment (control). 

As can be observed, none of the aqueous and ethanolic extracts showed any cytotoxic effect against normal lung cells (Hs888Lu). Previously, the selected plants were evaluated for the cytotoxicity against some carcinoma lines: *P. minus* against HeLa cells [37], *A. paniculata* against MCF-7 and HCT-116 [38], *M. charantia* against A431 and HaCaT cells [39] and *S. crispus* against MCF-7, MDA-MB-231, PC-3 and DU-145 cells [40]. While, the present study has assessed for the first time the cytotoxic activity of selected Malaysian plants against the human normal lung fibroblast cell line Hs888Lu.

## 3. Experimental

### 3.1. Plant Collections and Extractions

Air-dried leaves of selected Malaysian plants, namely *Polygonum minus* (kesum), *Andrographis paniculata* (hempedu bumi)*, Curcuma xanthorrhiza* (temulawak), *Momordica charantia* (bitter melon) and *Strobilanthes crispus* (pecah kaca), were obtained from Ethno Resources Sdn Bhd, Selangor, Malaysia, and identified by comparison with the Voucher specimen deposited at the Herarium of Rimba Ilmu, Institute of Science Biology and University of Malaya Kuala Lumpur.

### 3.2. Preparation of Aqueous Extract

The dried leaves of the selected plants were grounded into powder using an electrical blender followed by extraction with distilled water. Blended powders (50 g) were weighed and placed into a 1,000 mL flask. The distilled water was added in a 1:20 ratio. Subsequently it was heated and stirred on a hotplate for 3 h (65 °C) followed by cooling and filtration using filter paper and filter funnel, then distillated under reduced pressure in an Eyela rotary evaporator (Sigma-Aldrich, USA) until excess water was evaporated and finally subjected to lyophilization in a freeze-dryer to produce powdered forms of the extracts [41].

### 3.3. Preparation of Ethanol Extract

The dried powdered leaves were extracted by drenching in ethanol (100 g/2,000 mL, w/v) in a conical flask for 3 days at room temperature. Afterwards, the solvent was filtered, followed by distillation under reduced pressure in an Eyela rotary evaporator until excess solvent was evaporated [42].

### 3.4. DPPH Radical Scavenging Activity Assay

The antioxidant activity of the aqueous and ethanol extract were determined using 1,1-diphenyl-2-picrylhydrazyl (DPPH) radical base on the electron transfer reaction between DPPH reagent and the plant extracts. According to the method described by Gorinstein [34,43] with minor modification, stock solution (1 mg/1 mL) of the plant extracts and antioxidant standard (gallic acid) was prepared and then diluted to get five different concentrations. A quantity of each plant extract (5 µL) and standards were mixed with DPPH (195 µL). The mixture was then incubated at 37 °C for 30 min. The absorbance value was measured spectrophotometrically by a UV 1601 spectrophotometer (Shimadzu-Japan) at 517 nm.

### 3.5. Ferric Reducing Antioxidant Power Assay (FRAP)

FRAP was performed according to the method of and Erel [44]. Briefly, FRAP reagent was prepared at 37 °C from 300 mmol/L acetate buffer (pH 3.6), 10 mmol/L 2,4,6-tri[2-pyridyl]-*s*-triazine (TPTZ) in 40 mmol/L HCl, and 20 mmol/L FeCl_3_ in the ratio of 1:1:10. Ferrous sulfate heptahydrate (FeSO_4_·7H_2_O) was used as a standard and TPTZ working reagent used as a blank solution. The sample solution (10 µL, 1 mg/mL of plant extract), and the standards were mixed with FRAP reagents (300 µL). The mixture was then incubated at 37 °C and the absorbance was measured at 593 nm spectrophotometrically at 0 to 4 min. Finally, the values were expressed as mmol of gallic acid equivalents per 1 mg extract.

### 3.6. Total Phenolic Content (TPC)

The total phenolic compounds of aqueous and ethanol plant extracts were determined by the Folin-Ciocalteu method [45] with slight modifications. Standard solution was prepared by dissolving gallic acid (0.2 mg) in methanol (1 mL) then diluting with ddH_2_O to prepare different concentrations. The standards (10 µL), plant extracts (1 mg/1 mL DMSO) and positive control (quercetin at 1 mg/1 mL in DMSO) were added to Folin-Ciocalteu reagent (500 µL). After 5 minutes, 115g/L Na_2_CO_3_ solution (300 µL) was added to the mixture and thoroughly mixed. The mixtures were incubated at room temperature for 2 hr then the absorbance was measured at 765 nm using the UV 1601 spectrophotometer. Total phenolic content of the samples were determined and the amounts of phenolic compounds in plant extracts were expressed in mg/g of extract, gallic acid equivalent (GAE).

### 3.7. Cytotoxicity Assay

The cytotoxic activity of aqueous and ethanol extract of five Malaysian plants were carried out, using Promega Cell Titer 96 AQ_ueous_ Non-Radioactive Cell Proliferation (MTS) assay [46]. In this study human normal lung fibroblast cell line (Hs888Lu) was purchased from American Type Culture Collection (ATCC, The Global Bioresource Centre, Manassas, VA, USA). The Hs888Lu cells were cultured in Dulbecco’s Modified Eagle’s Medium (DMEM, Sigma, USA) with high glucose content, 1% non-essential amino acids (PAA Laboratory GmbH, Austria), 2% L-glutamine (200 mM) (Sigma, USA), 1% penicillin/streptomycin (100 ×) (PAA Laboratories GmbH, Austria), 1% sodium pyruvate (1 mM) (Sigma-Aldrich, USA), and supplemented with 10% fetal bovine serum (FBS, PAA Laboratory GmbH, Austria). The Hs888Lu were maintained in an incubator (Contherm Scientific Ltd, New Zealand) at 37 °C in a 5% CO_2_ atmosphere with 95% humidity.

The cell lines (1 × 10^5^ cells/mL) were seeded in a 96-well plate, and incubated at 37 °C under 5% CO_2_ in a humidified atmosphere for 24 hr before the addition of plant extract. The diluted solute of each extract ranging from 100 µg to 500 µg were added to the culture plate in triplicates then incubated for 24 hr under the same conditions. Following the treatment 20 µL of the MTS reagent (pre-warmed to 37 °C) was added to each of the 96 wells then the plate incubated at 37 °C for 3 hr. The absorbance was recorded using Glomax multi detection system (Promega, USA) at 492 nm.

### 3.8. Statistical Analysis

The data were analyzed by Statistical Package Social Science (SPSS) version 17.0. One-way ANOVA were used to show the mean differences between all samples.

## 4. Conclusion

Base on the present result and previous study we can conclude that the extracting solvent markedly influenced the antioxidant and TPC activity of the five Malaysian plants *Polygonum minus, Andrographis paniculata, Curcuma xanthorrhiza, Strobilanthes crispus* and *Momordica charantia*. Obviously, there was a significant difference between ethanol and aqueous plant extracts; ethanol extracts were more prominent in antioxidant activity and TPC. The results also did not show any cytotoxicity against the Hs888Lu cell line. Since *P. minus* exhibited high antioxidant and TPC; it can be used as a natural antioxidant. Further study needed to identify the exact active compound underlying this high antioxidant activity.

## Figures and Tables

**Table 1:** Antioxidant properties and total phenolic content (TPC) of aqueous and ethanolic extract of five Malaysian plants. Each value represents mean ± SD.

Plants	Extract	DPPH %	FRAP (mmol/g)	TPC (mg/g)
*P. minus*	aqueous	81.88 ± 0.98	849.33 ± 0.32	55.5 ± 0.0021
	ethanol	89.5 ± 1.07	11220 ± 0.1	207 ± 0.011
*C. xanthorrhiza*	aqueous	62.3 ± 1.76	358.3 ± 0.06	24.3 ± 0.005
	ethanol	64.0 ± 1.64	2955 ± 0.04	88.0 ± 0.002
*A. paniculata*	aqueous	69.7± 6.87	343.33 ± 0.05	23.33 ± 0.001
	ethanol	58.1 ± 12.70	1182 ± 0.01	19.0 ± 0.001
*S. crispus*	aqueous	28.5± 14.53	150.3 ± 0.01	8.33 ± 0.00
	ethanol	14.5 ± 0.64	108 ± 0.01	5.00 ±0.002
*M. charantia*	aqueous	17.4 ± 4.55	61.33 ± 0.01	7.67 ± 0.001
	ethanol	28.2 ± 1.90	388 ± 0.00	8.00 ± 0.001
Gallic acid	-	88.8 ± 0.85	1216.67 ± .03	-
Quercetin	-	-	-	63 ± 0.002
